# Supplementary material for: AWD regulates timed activation of BMP signaling in intestinal stem cells to maintain tissue homeostasis
Source: Nat Commun. 2019 Jul 5;10:2988. doi: 10.1038/s41467-019-10926-2 (PMC6611797; doi:10.1038/s41467-019-10926-2)
Supplement: Supplementary file 1 — Supplementary Information [file 41467_2019_10926_MOESM1_ESM.pdf]

Supplementary Information for

AWD regulates timed activation of BMP Signaling in intestinal stem cells to maintain tissue homeostasis

Xiaoyu Tracy Cai[1], Hongjie Li[2], Abu Safyan[3,4,5], Jennifer Gawlik[4,5,6], George Pyrowolakis[4,5,7] and Heinrich Jasper[1,8,9]*

[1]Buck Institute for Research on Aging, 8001 Redwood Boulevard, Novato, CA 94945-1400,USA.

[2]Department of Biology and Howard Hughes Medical Institute, Stanford University, Stanford, CA 94305, USA.

[3]International Max Planck Research School for Molecular and Cellular Biology (IMPRS-MCB), Max Planck Institute of Immunobiology and Epigenetics, 79108 Freiburg, Germany.

[4]Institute for Biology I, Faculty of Biology, Albert-Ludwigs-University of Freiburg, 79104 Freiburg, Germany.

[5]Center for Biological Systems Analysis (ZBSA), Albert-Ludwigs-University of Freiburg, 79104 Freiburg, Germany.

[6]Spemann Graduate School of Biology and Medicine (SGBM), Albert-Ludwigs-University of Freiburg, 79104 Freiburg, Germany.

[7]Signalling Research Centre BIOSS and CIBSS, Albert-Ludwigs-University Freiburg, 79104 Freiburg, Germany.

[8]Immunology Discovery, Genentech, Inc., 1 DNA Way, South San Francisco, California 94080, USA.

[9]Leibniz Institute on Aging - Fritz Lipmann Institute, Jena, Germany, 07745.

*correspondence to: jasperh@gene.com, Phone: (415)-798-7095.

Supplementary Fig.1

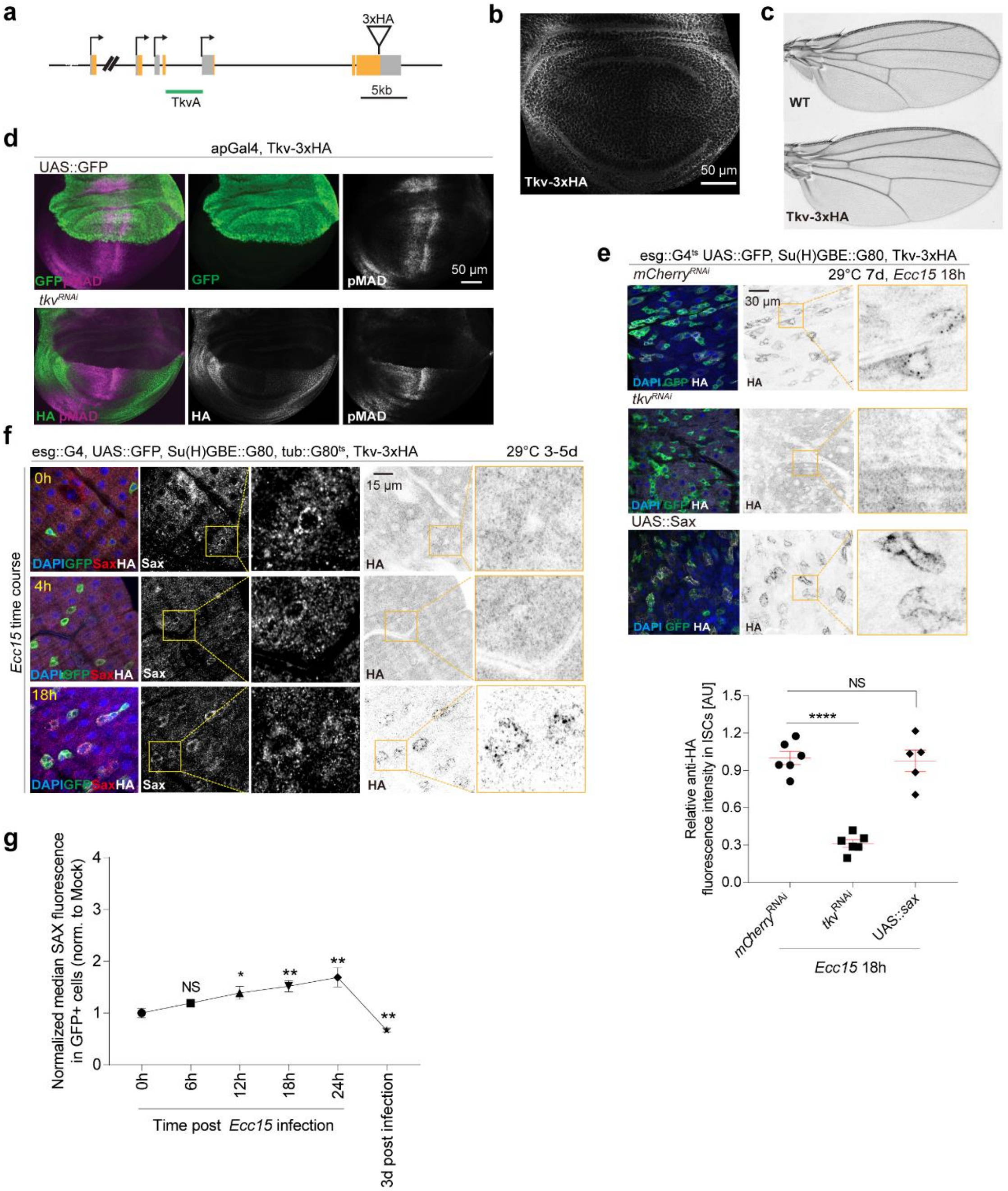

Supplementary Figure 1: Validation of Tkv-3xHA fly lines in larval imaginal disc and adult ISCs. (**a**) In scale illustration of the genome engineered *tkv* with exons shown as bars (open reading frames in orange, 5' and 3' UTR in grey). The genomic locus of *tkv* generates 4 different isoforms through alternative transcriptional start sites (arrows) resulting in proteins with different N-terminal sequences. The position and extent of TkvA are shown in green. The HA epitope tag was inserted just before the stop codon in the last exon of the gene, common to all isoforms. (**b**) Immunohistochemistry of Tkv-3xHA in a 3rd instar larval imaginal disc using an anti-HA antibody. Tkv-3xHA localizes at the plasma membrane and distributes in the typical tissue pattern with low levels of protein in medial and elevated levels in lateral regions of the disc. (**c**) Tkv-3xHA fly wings display no patterning and growth defects. (**d**) pMad staining in Tkv-3xHA fly wing imaginal discs expressing either GFP (control) or tkv^{RNAi} in dorsal cells under the control of apterous-Gal4 (apGal4). pMAD was dramatically reduced in dorsal cells when *tkv* was knocked down. (**e**) Tkv-3xHA expression level in ISCs expressing tkv^{RNAi} and ISCs overexpressing Sax after 18h *Ecc15* infection. (**f**) Different expression patterns of Sax and Tkv in ISCs during tissue regeneration. (**g**) Median fluorescence of Sax in GFP+ ISCs during the course of a 24h *Ecc15* infection, as measured by intracellular Flow Cytometry analysis. Fluorescence was normalized to the median value of control samples at 0h collected on the same day of measurement. Error bars indicate SEM (**e**: n=5-6 flies, **g**: n≥189 cells for each biological replicate). P values from Student's t-test (in **e**) or from one-tailed Wilcoxon rank-sum test (in **g**): ****p<0.0001; **p<0.01; *p<0.05; NS=not significant. One representative image from 4-8 flies tested in a single experiment was shown in **b**-**f**. Experiments were reproduced twice (in **b-f**) and at least 4 times (in **g**).

Supplementary Fig.2

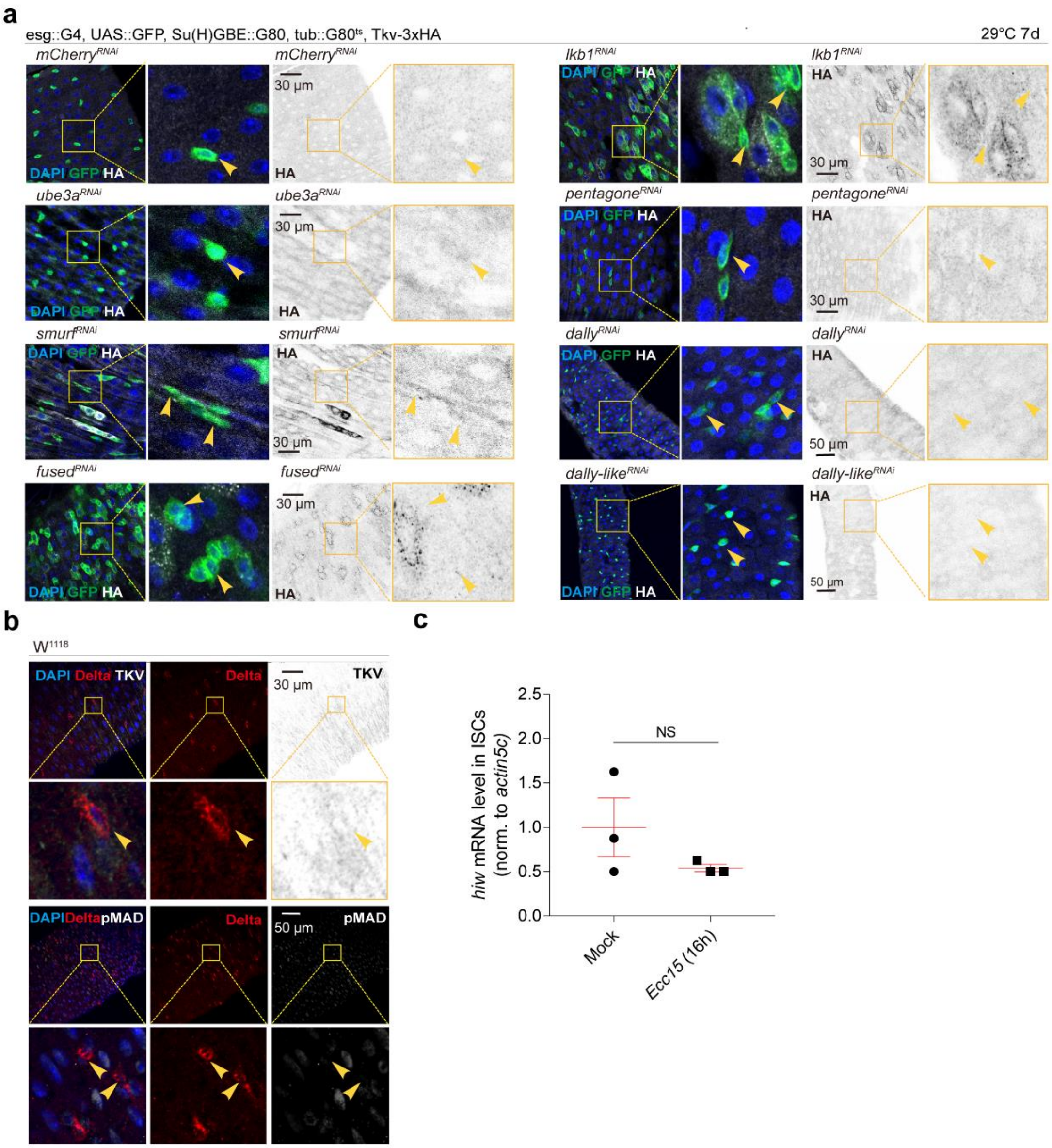

Supplementary Figure 2: A targeted RNAi screen to identify possible regulators of Tkv stability in ISCs. (**a**) Knocking down candidates: *Ube3a*, *Smurf*, *Fused*, *Lkb1*, *Dally*, *Dally-like*, *Pentagone*, didn't significantly induce Tkv-3xHA expression in ISCs. (**b**) Expression of Tkv and pMAD in wildtype ISCs (Delta+) of W^{1118} flies. (**c**) Relative mRNA levels of *highwire*, normalized to *actin5c*, in ISCs under homeostatic conditions and at 16h post-*Ecc15* infection. Error bars indicate SEM (n=3). P values from Student's t-test: NS=not significant. One representative image from 4-7 flies tested in a single experiment was shown in **a** and **b**. Experiments were reproduced twice.

Supplementary Fig.3

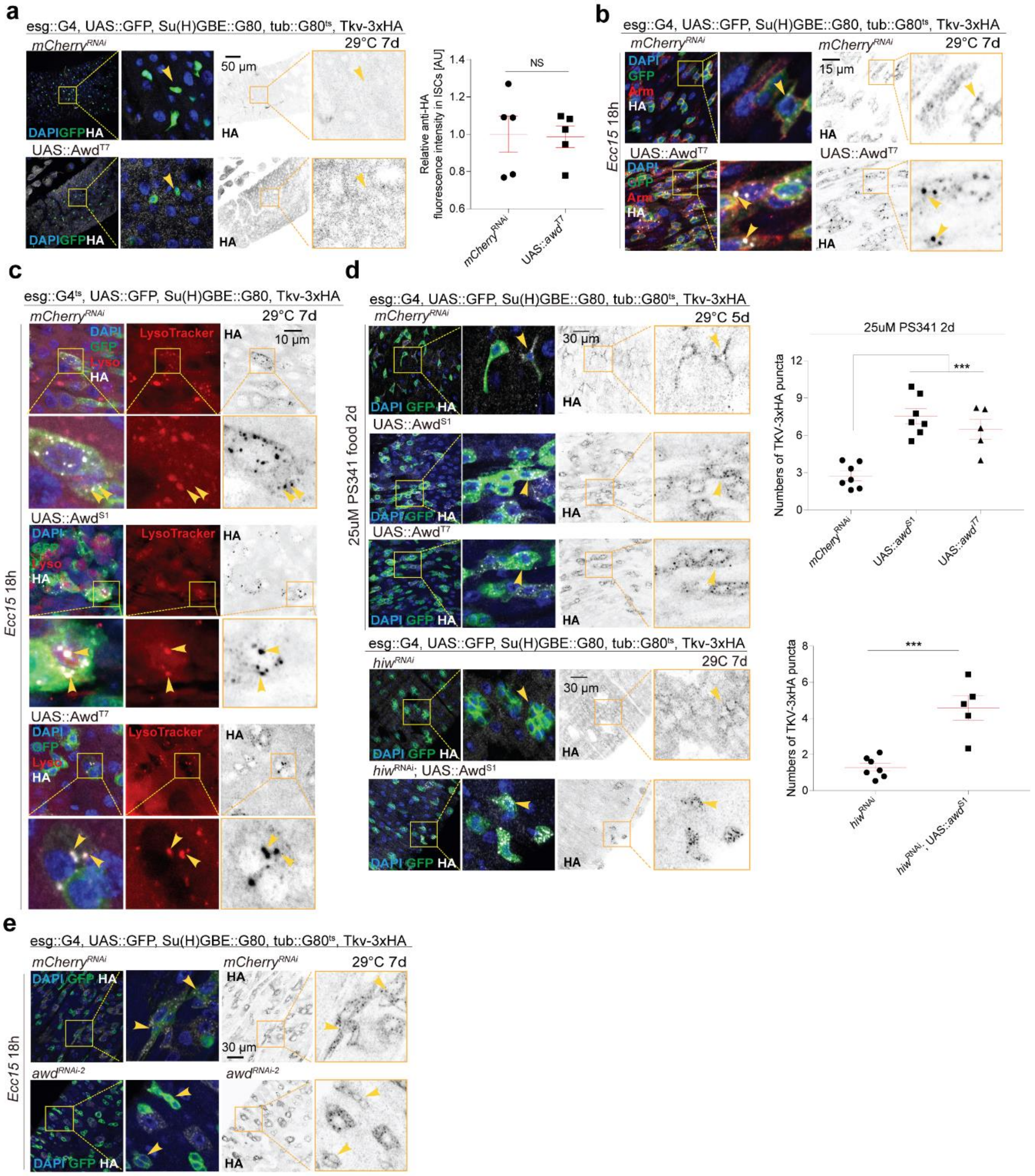

Supplementary Figure 3: AWD promotes internalization of Tkv. (**a**) Expression of Tkv-3xHA in wildtype and *awd*^{OE} ISCs (**a**: arrowheads, GFP+) under homeostatic conditions. (**b**) Expression of Tkv-3xHA in wildtype and *awd*^{OE} ISCs (**b**: arrowheads, GFP+) at 18h post-*Ecc15* infection. (**c**) Co-staining of lysosomes by LysoTracker and Tkv-3xHA in ISCs after 18h of *Ecc15* challenges, with and without *awd*^{OE}. (**d**) Tkv-3xHA expression in wildtype or *awd*^{OE} ISCs, when proteasome activity was inhibited by 25uM PS341 oral feeding for 2 days or when *highwire* was specifically knocked down in ISCs. (**e**) Tkv-3xHA expression in ISCs after 18h of *Ecc15* infection with a different *awd*^{RNAi} line overexpressed. P values from Student's t-test: ***p<0.001; NS=not significant. Experiments were repeated three times.

Supplementary Fig. 4

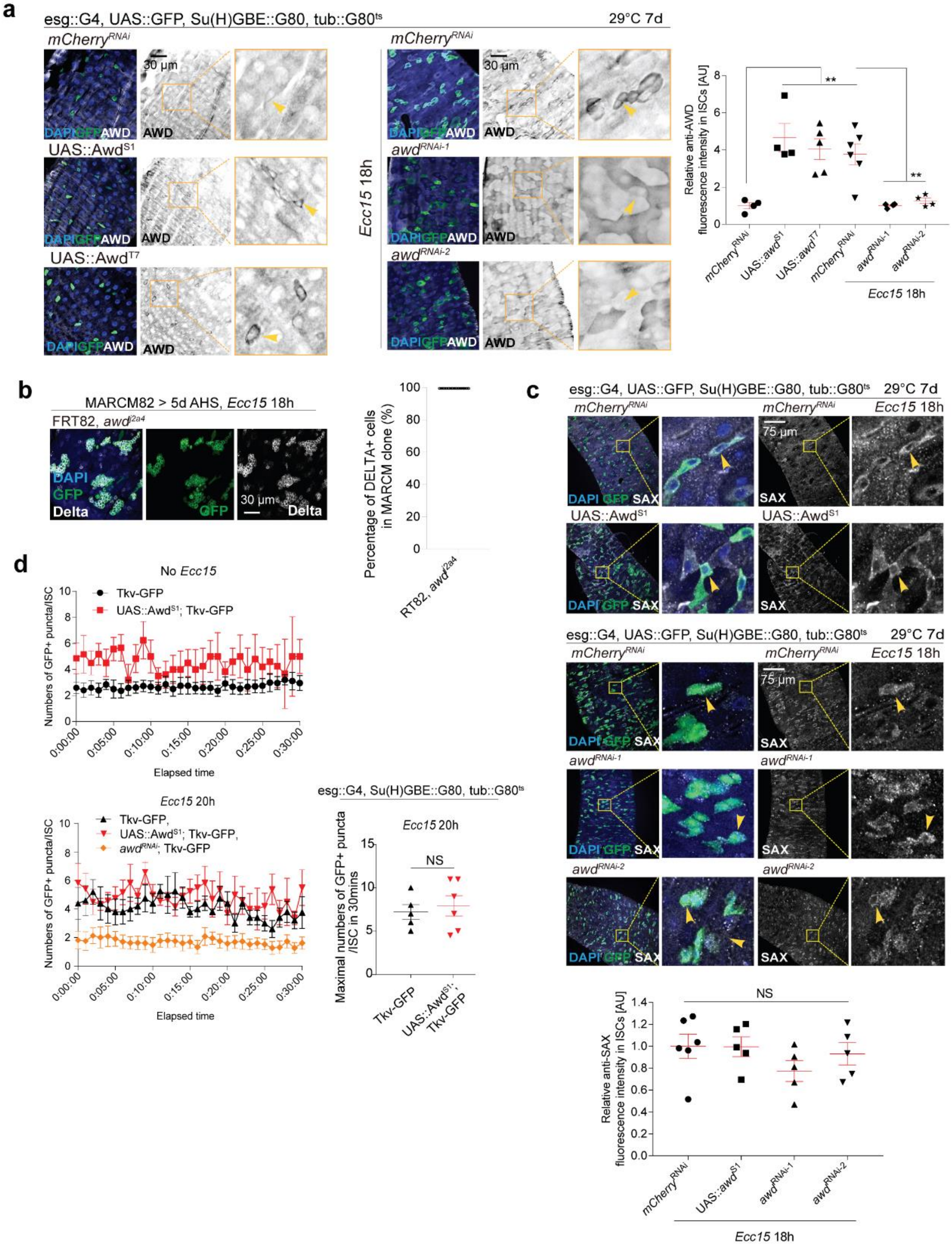

Supplementary Figure 4: AWD promotes internalization of Tkv, but not Sax. (**a**) Overexpression or knockdown efficiency of *awd* in ISCs was confirmed by immunohistochemistry using AWD antibody. (**b**) All the cells inside of *awd* mutant clones were DELTA+, quantified as average percentage of DELTA+ cells in MARCM clone per posterior midgut. (**c**) Immunostaining of SAX in ISCs (arrowheads, GFP+) upon *Ecc15* infection when *awd* was overexpressed or knocked down. (**d**) Analysis of 30min time-lapse movies with Tkv-GFP overexpressed in ISCs. Total numbers of Tkv-GFP puncta in ISCs at each time point within 30mins were quantified in wildtype, awd^{OE} and awd^{RNAi} ISCs with or without 20h of *Ecc15* infection. Error bars indicate SEM (**a**: n≥4 flies, **b**: n=7 flies, **c**: n=5-6 flies, **d**: n=5 flies). P values from Student's t-test: **p<0.01; NS=not significant. Experiments were repeated twice in **a**-**c**.

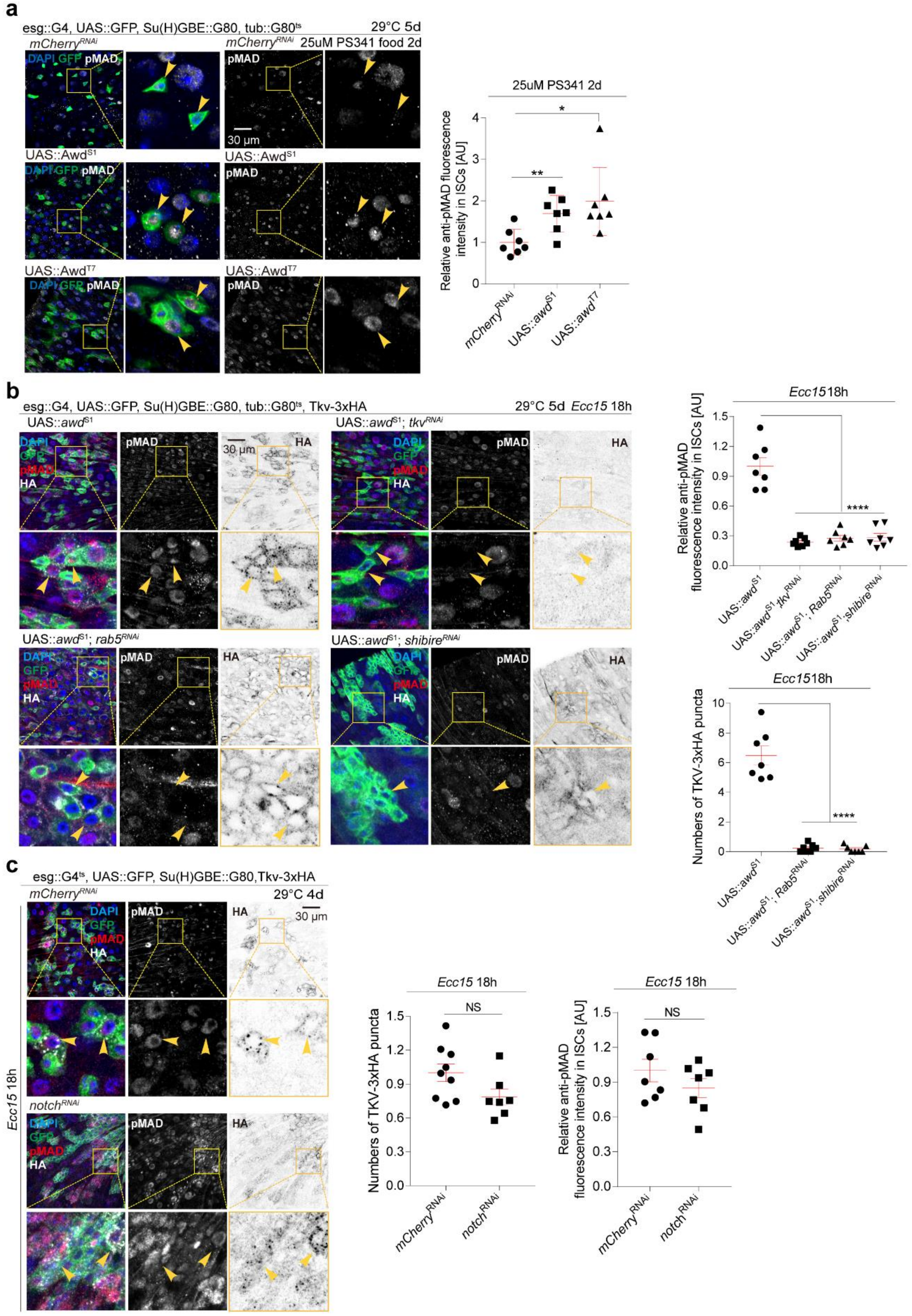
a
esg::G4, UAS::GFP, Su(H)GBE::G80, tub::G80ts
29°C 5d
mCherryRNAi
mCherryRNAi 25uM PS341 food 2d
DAPI GFP pMAD
pMAD
30 µm
UAS::AwdS1
UAS::AwdT7
25uM PS341 2d
Relative anti-pMAD fluorescence intensity in ISCs [AU]
mCherryRNAi
UAS::awdS1
UAS::awdT7
b
esg::G4, UAS::GFP, Su(H)GBE::G80, tub::G80ts, Tkv-3xHA
29°C 5d Ecc15 18h
UAS::awdS1
UAS::awdS1; tkvRNAi
UAS::awdS1; rab5RNAi
UAS::awdS1; shibireRNAi
DAPI
GFP
pMAD
HA
Ecc15 18h
Relative anti-pMAD fluorescence intensity in ISCs [AU]
****
UAS::awdS1
UAS::awdS1;tkvRNAi
UAS::awdS1; Rab5RNAi
UAS::awdS1;shibireRNAi
Numbers of TKV-3xHA puncta
c
esg::G4ts, UAS::GFP, Su(H)GBE::G80,Tkv-3xHA
mCherryRNAi
29°C 4d
30 µm
Ecc15 18h
notchRNAi
NS
Numbers of TKV-3xHA puncta
Relative anti-pMAD fluorescence intensity in ISCs [AU]
mCherryRNAi
notchRNAi

Supplementary Figure 5: Regulation of MAD signaling by AWD is Rab5 and Dynamin(Shibire) dependent. (**a**) Expression of pMAD in ISCs (arrowheads, GFP+) in which proteasome activity was inhibited by 2day feeding of 25 μM PS341, with or without *awd* overexpression. (**b**) Expression of Tkv-3xHA and pMAD in wildtype and *notch*^{RNAi} ISCs (arrowheads, GFP+) at 18h post-*Ecc15* infection. (**c**) Expression of Tkv-3xHA and pMAD in *awd*^{OE} ISCs (arrowheads, GFP+) at 18h of *Ecc15* infection when *tkv*^{RNAi}, *rab5*^{RNAi} or *shibire*^{RNAi} was co-expressed respectively. Error bars indicate SEM (**a**-**c**: n=7 flies). P values from Student's t-test: ****p<0.0001; **p<0.01; *p<0.05; NS=not significant. Experiments were repeated three times.

Supplementary Fig. 6

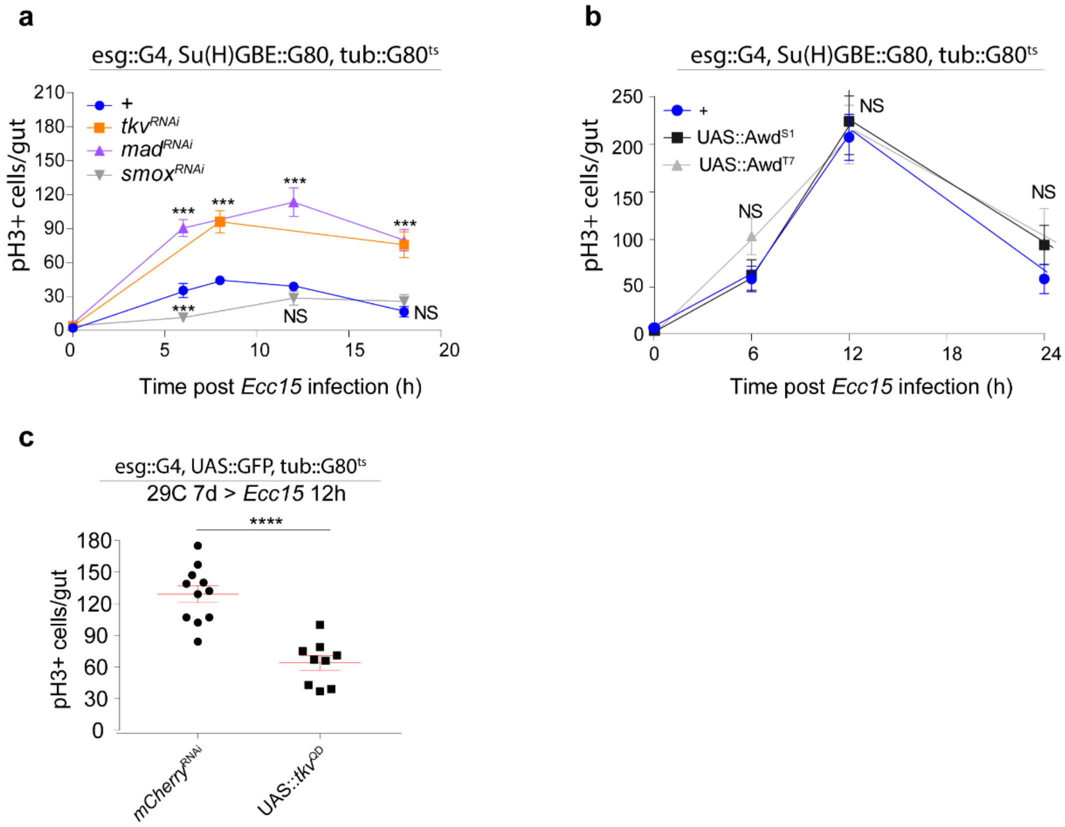

Supplementary Figure 6: AWD is not sufficient to inhibit ISC proliferation. (**a, b**) Dynamic mitotic activity of ISCs, when *tkv, mad* or *smox* was respectively knocked down (**a**), or when *awd* was overexpressed, measured as numbers of pH3+ cells per gut during the course of an *Ecc*15 infection episode up to 24h. (**c**) Mitotic activity of ISCs with or without Tkv^{QD} overexpression in response to 12h *Ecc15* infection, measured as numbers of pH3+ cells per gut. Error bars indicate SEM (**a**: n≥10 flies, **b**: n≥10 flies, **c**: n≥9 flies). P values from Student's t-test: ****p<0.0001; ***p<0.001; NS=not significant. Experiments were repeated twice.

Supplementary Fig. 7

Example of Flow Cytometry analysis

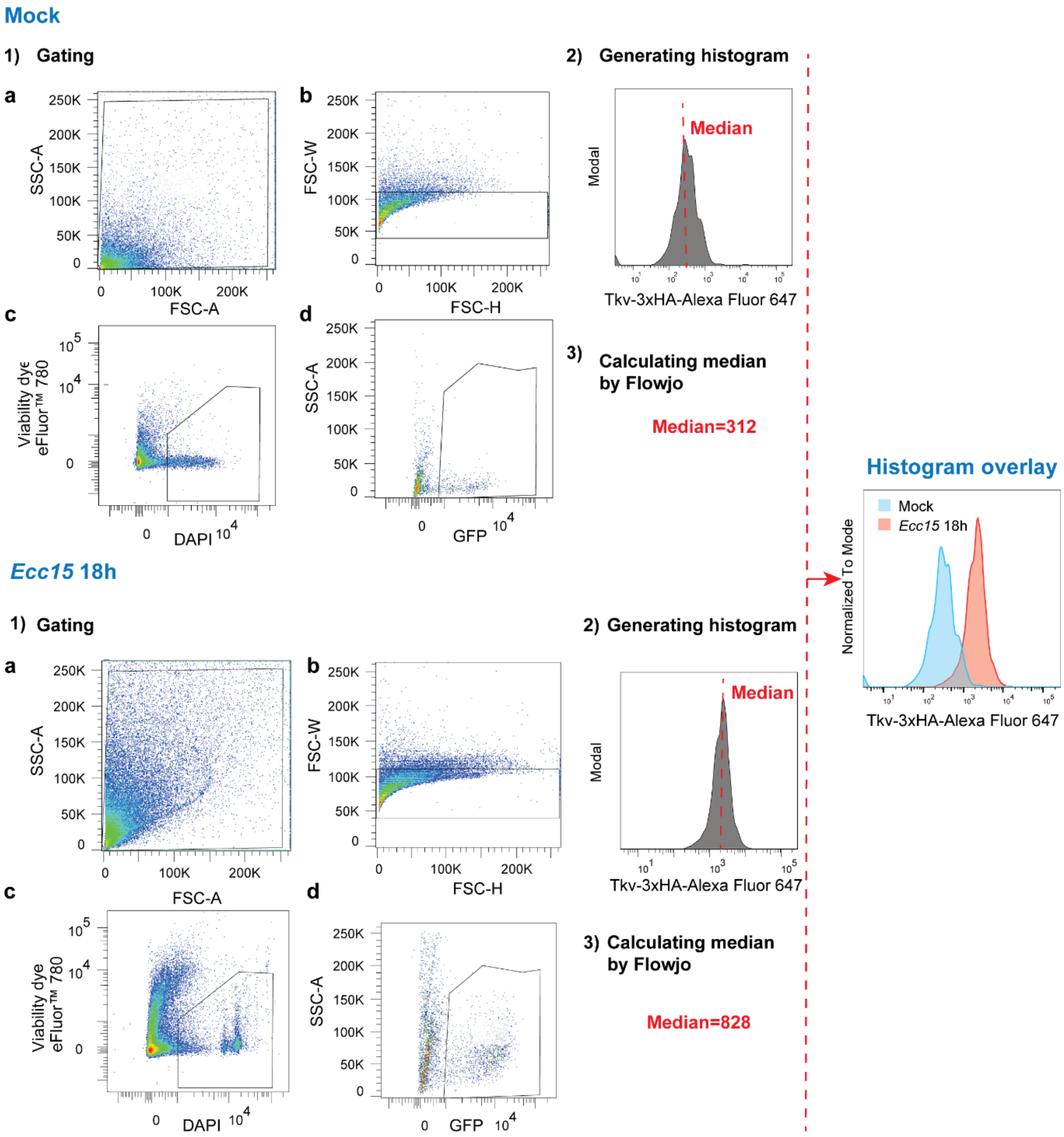

Supplementary Figure 7: Example of intracellular Flow Cytometry analysis. Single cell suspension from whole guts of flies fed with *Ecc15* or 5% sucrose (mock) for 18 hours, as described in the method, were loaded into BD Symphony flow cytometer. GFP-labelled ISCs were sorted by the following gates: forward versus side scatter (FSC vs SSC, **a**), forward scatter height versus width (FSC-H vs FSC-W, **b**), fixable viability dye (eFluor™ 780 to label dead cells before fixation) versus DAPI (labelling nuclei to exclude debris) (**c**), and side scatter versus GFP fluorescence channel (SSC vs GFP, **d**). A histogram was generated for each sample, with the Tkv-3xHA fluorescence intensity level as the x-axis (logarithmic scale) and the number of events (normalized to its peak height) as the y-axis. Median fluorescence intensity of Tkv-3xHA staining for each sample was computed by FlowJo software and used to compare the difference of Tkv-3xHA expression level in ISCs between samples. Measurement of Tkv-3xHA (Fig.1f, 2e), Sax (Supplementary Fig. 1g), Highwire (Fig. 2g), AWD (Fig. 3b) and pMAD (Fig. 5b, 5d, 5f) expression levels in GFP+ ISCs under conditions as noted were accomplished with the same gating strategy applied.
