# Supplementary material for: AWD regulates timed activation of BMP signaling in intestinal stem cells to maintain tissue homeostasis
Source: Nat Commun. 2019 Jul 5;10:2988. doi: 10.1038/s41467-019-10926-2 (PMC6611797; doi:10.1038/s41467-019-10926-2)
Supplement: Supplementary file 3 — Reporting Summary [file 41467_2019_10926_MOESM3_ESM.pdf]

nature research

Corresponding author(s): Heinrich Jasper

Last updated by author(s): Apr 1, 2019

Reporting Summary

Nature Research wishes to improve the reproducibility of the work that we publish. This form provides structure for consistency and transparency in reporting. For further information on Nature Research policies, see Authors & Referees and the Editorial Policy Checklist.

Statistics

For all statistical analyses, confirm that the following items are present in the figure legend, table legend, main text, or Methods section.

n/a	Confirmed	
☐	☒	The exact sample size (*n*) for each experimental group/condition, given as a discrete number and unit of measurement
☐	☒	A statement on whether measurements were taken from distinct samples or whether the same sample was measured repeatedly
☐	☒	The statistical test(s) used AND whether they are one- or two-sided<br>*Only common tests should be described solely by name; describe more complex techniques in the Methods section.*
☒	☐	A description of all covariates tested
☐	☒	A description of any assumptions or corrections, such as tests of normality and adjustment for multiple comparisons
☐	☒	A full description of the statistical parameters including central tendency (e.g. means) or other basic estimates (e.g. regression coefficient) AND variation (e.g. standard deviation) or associated estimates of uncertainty (e.g. confidence intervals)
☐	☒	For null hypothesis testing, the test statistic (e.g. F, t, r) with confidence intervals, effect sizes, degrees of freedom and P value noted<br>*Give P values as exact values whenever suitable.*
☒	☐	For Bayesian analysis, information on the choice of priors and Markov chain Monte Carlo settings
☒	☐	For hierarchical and complex designs, identification of the appropriate level for tests and full reporting of outcomes
☒	☐	Estimates of effect sizes (e.g. Cohen's d, Pearson's r), indicating how they were calculated

Our web collection on statistics for biologists contains articles on many of the points above.

Software and code

Policy information about availability of computer code

Data collection	No software was used
Data analysis	Data was analyzed in GraphPad Prism v7.02 and MS Excel software

For manuscripts utilizing custom algorithms or software that are central to the research but not yet described in published literature, software must be made available to editors/reviewers. We strongly encourage code deposition in a community repository (e.g. GitHub). See the Nature Research guidelines for submitting code & software for further information.

Data

Policy information about availability of data

All manuscripts must include a data availability statement. This statement should provide the following information, where applicable:

- Accession codes, unique identifiers, or web links for publicly available datasets
- A list of figures that have associated raw data
- A description of any restrictions on data availability

The authors declare that the data supporting the findings of this study are available within the paper and its supplementary information files.

Field-specific reporting

Please select the one below that is the best fit for your research. If you are not sure, read the appropriate sections before making your selection.

☒ Life sciences ☐ Behavioural & social sciences ☐ Ecological, evolutionary & environmental sciences

For a reference copy of the document with all sections, see nature.com/documents/nr-reporting-summary-flat.pdf

Life sciences study design

All studies must disclose on these points even when the disclosure is negative.

Sample size	No sample size calculation was done. Numbers used were described in the figure legends.
Data exclusions	N/A
Replication	Experiments in this study were repeated three times or twice, as noted in the figure legends.
Randomization	All experiments were done using a pool of siblings hatched at same times and raised under same conditions. No randomization was performed among differentially treated animals.
Blinding	No blinding was done.

Reporting for specific materials, systems and methods

We require information from authors about some types of materials, experimental systems and methods used in many studies. Here, indicate whether each material, system or method listed is relevant to your study. If you are not sure if a list item applies to your research, read the appropriate section before selecting a response.

Materials & experimental systems

n/a	Involved in the study	
☐	☒	Antibodies
☒	☐	Eukaryotic cell lines
☒	☐	Palaeontology
☐	☒	Animals and other organisms
☒	☐	Human research participants
☒	☐	Clinical data

Methods

n/a	Involved in the study	
☒	☐	ChIP-seq
☐	☒	Flow cytometry
☒	☐	MRI-based neuroimaging

Antibodies

Antibodies used	Primary antibodies and dilution used in Immunostaining: rabbit anti-pSMad3 (Epitomics Cat. No. EP823Y, 1:500), rabbit anti-β-galactosidase (Cappel MP Biomedicals, 1:5000), rabbit anti-phospho-Histone H3 Ser 10 (EMD Millipore Cat. No. 06-570, 1:1000), rabbit anti-AWD (gift from Dr. Tien Hsu, 1:100), mouse anti-Armadillo (DSHB, 1:100), mouse anti-Prospero (DSHB, 1:50), rabbit anti-Sax (Abcam Cat. No. ab42105, 1:200), rat anti-Delta (gift from Dr. Matthew D. Rand, 1:1000), rabbit anti-Tkv (gift from Dr. Marcos Gonzalez-Gaitan, 1:100), rat anti-HA (Roche Cat. No. 11867423001, dissolved in distilled water and stored at 100ug/ml, 1:300), rabbit anti-HA (Cell Signaling Cat. No. 3724S, 1:100).<br><br>Primary antibodies used in Flow Cytometry: : rabbit anti-pSMad3 (Epitomics Cat. No. EP823Y, 1:800), rat anti-HA (Roche Cat. No. 11867423001, dissolved in distilled water and stored at 100ug/ml, 1:500), rabbit anti-Sax (Abcam Cat. No. ab42105, 1:500), rabbit anti-AWD (gift from Dr. Tien Hsu, 1:300), mouse anti-Highwire (DSHB, 6H4, 1:300).<br><br>Fluorescent secondary antibodies were bought from Jackson Immunoresearch (1:500).
Validation	Rabbit anti-AWD antibody (a gift from Dr. Tien Hsu) was validated in Supplementary Fig.4a. Validation for other antibodies was done previously by us and others, as these antibodies are commonly used and published antibodies.

Animals and other organisms

Policy information about studies involving animals; ARRIVE guidelines recommended for reporting animal research

Laboratory animals	For all studies we used adult females Drosophila melanogaster. Full genotypes were labelled for all figures.
Wild animals	N/A
Field-collected samples	N/A
Ethics oversight	N/A

Note that full information on the approval of the study protocol must also be provided in the manuscript.

Flow Cytometry

Plots

Confirm that:

☒ The axis labels state the marker and fluorochrome used (e.g. CD4-FITC).

☒ The axis scales are clearly visible. Include numbers along axes only for bottom left plot of group (a 'group' is an analysis of identical markers).

☐ All plots are contour plots with outliers or pseudocolor plots.

☒ A numerical value for number of cells or percentage (with statistics) is provided.

Methodology

Sample preparation	GFP-labelled ISCs were freshly prepared for each replicate, from 15-20 guts, as described in the methods.
Instrument	BD Symphony flow cytometer.
Software	FlowJo v10 Software
Cell population abundance	Cell counts for each population were described in figure legends.
Gating strategy	Forward versus side scatter (FSC vs SSC); forward scatter height versus width (FSC-H vs FSC-W); fixable viability dye (eFluor™ 780 to label dead cells before fixation) versus DAPI (labeling nuclei to exclude debris); side scatter versus GFP fluorescence channel (SSC vs GFP).

☒ Tick this box to confirm that a figure exemplifying the gating strategy is provided in the Supplementary Information.
