# Supplementary material for: AWD regulates timed activation of BMP signaling in intestinal stem cells to maintain tissue homeostasis
Source: Nat Commun. 2019 Jul 5;10:2988. doi: 10.1038/s41467-019-10926-2 (PMC6611797; doi:10.1038/s41467-019-10926-2)
Supplement: Supplementary file 4 — Description of Additional Supplementary Files [file 41467_2019_10926_MOESM4_ESM.pdf]

Description of Additional Supplementary Files

Supplementary Movie 1: Tkv-GFP presents as puncta in ISCs upon *Ecc15* infection, related to Figure 1. Flies with ISCs overexpressing Tkv-GFP (esgG4, Su(H)GBEG80, tubG80^{ts}, green) for 6 days were orally fed with *Ecc15* for 20 hours before dissection and imaged *ex vivo* at 30s intervals for 45mins. Timelapse indicated as minutes:seconds.

Supplementary Movie 2: Dynamics of Tkv-GFP puncta and lysosomes in wildtype ISCs under homeostatic conditions, related to Figure 3 and Supplementary Figure 4. ISCs overexpressing Tkv-GFP (esgG4, Su(H)GBEG80, tubG80^{ts}, green) for 7 days were imaged *ex vivo* at 30s intervals for 30mins. Lysosomes were indicated by LysoTracker staining (red). Timelapse indicated as minutes: seconds.

Supplementary Movie 3: Dynamics of Tkv-GFP puncta and lysosomes in *awd*^{OE} ISCs under homeostatic conditions, related to Figure 3 and Supplementary Figure 4. Flies with ISCs co-expressing *awd*^{OE} and Tkv-GFP (esgG4, Su(H)GBEG80, tubG80^{ts}, green) for 7 days were imaged *ex vivo* at 30s intervals for 30mins. Lysosomes were indicated by LysoTracker staining (red). Timelapse indicated as minutes: seconds.

Supplementary Movie 4: Dynamics of Tkv-GFP puncta and lysosomes in wildtype ISCs upon *Ecc15* infection, related to Figure 3 and Supplementary Figure 4. Flies with ISCs overexpressing Tkv-GFP (esgG4, Su(H)GBEG80, tubG80^{ts}, green) for 6 days were orally fed with Ecc15 for 20 hours before dissection and imaged ex vivo at 30s intervals for 30mins. Lysosomes were indicated by LysoTracker staining (red). Timelapse indicated as minutes: seconds.

Supplementary Movie 5: Dynamics of Tkv-GFP puncta and lysosomes in *awd*^{OE} upon *Ecc15* infection, related to Supplementary Figure 4. Flies with ISCs co-expressing *awd*^{OE} and Tkv-GFP (esgG4, Su(H)GBEG80, tubG80^{ts}, green) for 6 days were orally fed with *Ecc15* for 20 hours before dissection and imaged *ex vivo* at 30s intervals for 30mins. Lysosomes were indicated by LysoTracker staining (red). Timelapse indicated as minutes: seconds.

Supplementary Movie 6: Dynamics of Tkv-GFP puncta and lysosomes in *awd*^{RNAi} upon *Ecc15* infection, related to Figure 3 and Supplementary Figure 4. Flies with ISCs co-expressing *awd*^{RNAi} and Tkv-GFP (esgG4, Su(H)GBEG80, tubG80^{ts}, green) for 6 days were orally fed with *Ecc15* for 20 hours before dissection and imaged *ex vivo* at 30s intervals for 30mins. Lysosomes were indicated by LysoTracker staining (red). Timelapse indicated as minutes: seconds.
